# Lactoferrin: A Modulator for Immunity against Tuberculosis Related Granulomatous Pathology

**DOI:** 10.1155/2015/409596

**Published:** 2015-12-14

**Authors:** Jeffrey K. Actor

**Affiliations:** ^1^UTHealth, Department of Pathology, University of Texas-Houston Medical School, Houston, TX 77030, USA; ^2^Program in Immunology, Graduate School of Biomedical Sciences, Houston, TX 77030, USA

## Abstract

There is great need for a therapeutic that would limit tuberculosis related pathology and thus curtail spread of disease between individuals by establishing a “firebreak” to slow transmission. A promising avenue to increase current therapeutic efficacy may be through incorporation of adjunct components that slow or stop development of aggressive destructive pulmonary pathology. Lactoferrin, an iron-binding glycoprotein found in mucosal secretions and granules of neutrophils, is just such a potential adjunct therapeutic agent. The focus of this review is to explore the utility of lactoferrin to serve as a therapeutic tool to investigate “disruption” of the mycobacterial granuloma. Proposed concepts for mechanisms underlying lactoferrin efficacy to control immunopathology are supported by data generated based on* in vivo* models using nonpathogenic trehalose 6,6′-dimycolate (TDM, cord factor).

## 1. Introduction

There is great need for a therapeutic that would limit tuberculosis pathology and thus limit spread of disease between individuals by establishment of a “firebreak” to slow transmission [1]. A promising avenue to increase current therapeutic efficacy may be through incorporation of adjunct components that curtail development of aggressive destructive pulmonary pathology. Lactoferrin, an iron-binding glycoprotein found in mucosal secretions and granules of neutrophils, is just such a potential adjunct therapeutic agent. The focus of this review is therefore to explore the utility of lactoferrin to serve as a therapeutic tool to investigate “disruption” of the mycobacterial granuloma during infection. In addition, mechanisms for lactoferrin efficacy will be proposed based on manipulation of trehalose 6,6′-dimycolate (TDM, cord factor) immunopathology developed in a nonpathogenic* in vivo* setting. The studies here indicate that lactoferrin may be a useful adjunct therapeutic for amelioration of pathological response to mycobacteria.

*Mycobacterial Granulomas*. Primary Tuberculosis (TB) begins with infection that spreads via lymphatics and blood stream before inducing systemic immunity that contains and controls the organisms within granulomas. Induction of the* Mycobacterium tuberculosis* (MTB) disease-induced granuloma is dependent on factors involved in response initiation and associated immune activity [2–7]. In the most extreme simplification of the process, the underlying initiator is the existence of a poorly degradable persistent antigenic source. Mycobacterial organisms survive within macrophages, exhibiting slow release of antigens to recruited cells. The net result of granuloma formation is of initial benefit to both the host and the organism, allowing control of infection while providing a place for organisms to hide until time for expansion and subsequent transmission to other individuals [8, 9]. In reality, the organism exhibits effects on tissues surrounding infected cells through released potent, bioactive cell wall constituents [10]. New paradigms implicate inflammatory processes beginning with induction of a lipid-related necrotic pneumonia that may transition to become the focus of granulomas and fibrocaseous disease [11–14]. Regardless of event initiation, it is clear that over time the disruptive nature of the granuloma causes harm to the infected host.

Our laboratory has studied granuloma pathobiology in detail in mouse models of tuberculosis [8, 15–20] and made contributions to factors involved in the development of progressive pathology. Furthermore, we utilized our knowledge to design protocols to maximize immune-modulating agents to investigate the relationship of protection and pathology [21–24]. An area of study used lactoferrin as an immune modulator to augment the BCG vaccine and relative responses to mycobacterial antigens [22–27], leading to reduction in pathological damage after challenge with virulent organisms. They were the observations made during development of human lactoferrin as a vaccine adjuvant that led us to believe it also had utility as an immune modulator to reduce inflammation during granuloma formation [23, 28].

Many of the molecular mechanisms involved in development, maintenance, and initiation of factors related to mycobacterial pathology in the lung have been identified. A system of choice is a model based on the noninfectious proinflammatory granuloma induced by the major surface glycolipid from pathogenic organisms, developed by multiple investigators, including Retzinger, Behling, and Hunter [29–32]. This has permitted a global understanding of the immunopathology. Injection with cord factor (trehalose 6,6′-dimycolate, TDM) induces transient pulmonary granulomas that increase in number and complexity over a 7-day period, followed by resolution of pathology [33]. Manipulation of host factors has allowed definition of immune parameters contributing to the initiation, maintenance, and resolution phases of response. 

*The Cord Factor Model of the Mycobacterial Granuloma*. Cord factor (trehalose 6,6′-dimycolate, TDM) is the most abundant glycolipid produced on the surface of mycobacterial organisms. TDM plays multiple roles in the pathogenesis of MTB [34, 35], including the formation of caseation in the lung after infection [36]. TDM formulated into an emulsion, or placed on beads, and injected intravenously into mice induces a lung pathology that mimics many aspects of early MTB infection, including granulomatous response and the production of proinflammatory cytokines. The TDM model of granuloma formation has been used to elucidate the immunological factors involved in the granulomatous response [37, 38]. Furthermore, TDM can induce activated foreign body type granulomas in naïve mice [33, 39], and hypersensitivity (immune) granulomas in appropriately sensitized mice [36, 40, 41]. Therefore, this model system is ideal for exploring the potential of immunomodulators to alter granuloma structure, with perhaps specific utility to extrapolate findings to immune related pathology identified during clinical manifestation of tuberculosis disease.

**Figure 1:**
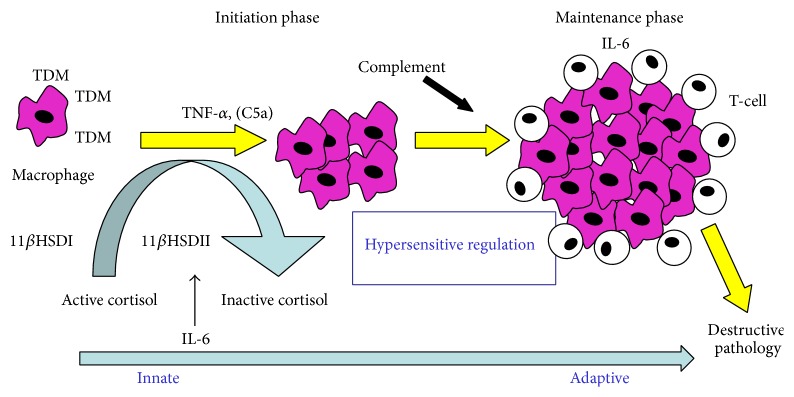
Mechanisms of TDM-induced granulomas. Cytokines and immune-regulatory factors induced by mycobacterial glycolipid TDM play key roles in the development of pathology induced during mycobacterial infection. Model systems using isolated TDM allow identification of host immune function to extrapolate findings of immune related pathology occurring during clinical manifestation of tuberculosis disease [17, 33, 37, 38, 41, 52–58].

The interactions of mycobacteria and its lipid components with cell surface antigens to elicit inflammatory responses have been characterized [42] and are the subject of other papers and reviews [43–46]. Recent discoveries link the C-type lectin mincle [47–49] as a prime candidate receptor for TDM. This is augmented in part by interactions with MARCO, TLR2, and/or CD14, all of which are critical to mediate activity [44]. These are combined with internalized signaling events that possibly function through Card9-dependent mechanisms [50, 51]. Suffice to say, TDM's interaction with surface “receptors” is critical for initiation of cellular activation of TLR and NOD pathways, as well as control of intracellular trafficking events [52, 53]. Using knockout mice, established patterns of cytokine production were found to be associated with pathology. This allowed the delineation of the major molecular events in innate establishment and maintenance of the TDM induced granuloma [17, 33, 37, 38, 41, 52–56]. A link was also established to adaptive (T-cell mediated) hypersensitive responses critical for development of pathological granulomas [57, 58] (Figure 1). 

*Lactoferrin as a First-Line Defense Protein*. Lactoferrin is a monomeric 80 kDa single polypeptide chain contained in most mammalian exocrine secretions, including milk, tears, saliva, and bronchial and intestinal secretions. It is also present in the secondary granules of neutrophils. It is considered a first-line defense protein involved in protection against microbial infections [59, 60] and subsequent development of systemic inflammatory response syndrome (SIRS) and sepsis [61–67]. More recently, lactoferrin has been implicated in immunoregulatory functions [62, 68–73], and modulation of vaccine function [22–27].

**Figure 2:**
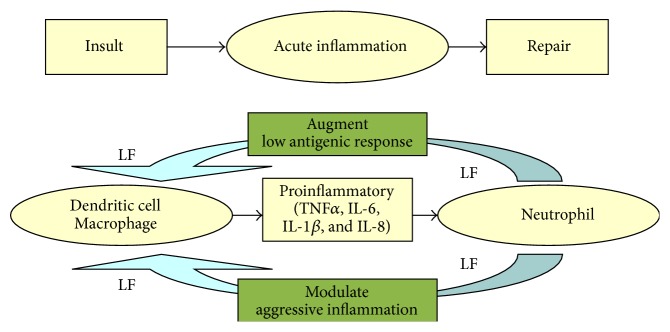
Lactoferrin mediation of insult-induced inflammation. Insult, defined as infection, trauma, or chronic illness, leads to activation of macrophage and dendritic cells. Activated neutrophils degranulate at the site of injury and release massive amounts of lactoferrin. Lactoferrin, in turn, can either augment low level responses or modulate overaggressive cytokine activity. Both effects serve to control inflammation to assist with tissue repair after insult (modified from [74]).

There are two primary forms of human lactoferrin, one contained in exocrine secretions and the other present in secondary granules of neutrophils. The two forms are identical in their amino acid sequence but differ in glycan content [75, 76]. While the secreted form is thought to be involved in the host defense against microbial infection at mucosal sites, granulocytic/neutrophilic lactoferrin has notable immunomodulatory function [74]. Neutrophilic lactoferrin is an integral part of the cytokine-mediated cascade during insult-induced metabolic imbalance [77–81] (Figure 2), with a subset of biological properties unique from lactoferrins produced at other sources by other cell phenotypes [82].

The key to understanding the molecular basis of its activities is thought to reside in both patterns of glycosylation and sialylation [83, 84]. The primary structure of human LF is characterized by a single polypeptide chain containing 692 amino acids organized in two highly homologous lobes, designated the N- and C-lobe, each capable of binding single ferric ion (Fe+++). Lactoferrin is a glycoprotein and in humans the glycans are the N-acetyllactosaminic type, *α*1,3-fucosylated on the N-acetyl-glucosamine residue linked to the peptide chain. There are three possible N-linked glycosylation sites in hLF, one at Asn138, a second site at Asn479, and a third site at Asn624; differential utilization of these sites results in distinct glycosylation variants [85]. Many observed activities of LF are dependent upon specific glycosylation patterns. For example, the immunoregulatory activity of LF in humans is dependent on the interaction of this glycoprotein with a receptor specific for sialic acid, and direct lymphocyte activation by LF requires sialylation [86].

## 2. Lactoferrin as an Immune Modulator

### 2.1. Lactoferrin: A Balance between Potentiation and Mediation

Lactoferrin bridges innate and adaptive immune functions by regulating specific target cell responses [87, 88]. By acting as a homeostatic modulator, lactoferrin can work both ends of the immunological spectrum to increase low response or dampen aggressive ones. It has direct capacity to regulate both proinflammatory and anti-inflammatory responses [62, 63]. The utility of such an immune mediator represents a novel therapeutic agent dependent on elicited responses for outcome [74]. A direct example of how lactoferrin can manipulate outcomes of response is exemplified by knowledge that lactoferrin can bind to the soluble CD14 and the CD14/LPS complex [89], an interaction which has been shown to mediate toll-like receptor-4 (TLR-4) pattern recognition events [90]. Indeed, lactoferrin mediated events related to TLR-4 may represent a strong strategy to block excessive antigen presenting cell activation [91]. What remains unknown is how lactoferrin might affect PAMP signaling processes during tuberculosis infection. While some data suggests that Myd88 pathways may not be as critical in transition to adaptive defense against tuberculosis [92], more recent studies suggest that subversion of the TLR-2-MyD88 pathway is an important factor in intracellular processing [93]. In either case, it would seem that lactoferrin may function as a regulator in an independent manner during immune modulation during TB challenge.

We and collaborators investigated lactoferrin to modify innate events during inflammation, such as those seen during systemic inflammatory response syndrome and models of oxidative stress [78, 94–97]. Our laboratory also spent years investigating lactoferrin augmentation of the BCG vaccine to protect against subsequent challenge with virulent MTB, functioning through strong induction of cell-mediated immunity [64, 98] and generation of IFN-*γ* antigen specific recall responses [24, 26, 27, 69, 98, 99]. Lactoferrin has a profound modulatory action on adaptive immune functions [70, 73] by promoting maturation of T-cell precursors into competent helper cells and differentiation of immature B-cells into efficient antigen presenting cells [100]. The innate and adaptive events are not mutually exclusive, but rather complementary, and lend credence to utility of lactoferrin for immune homeostasis in insult-induced metabolic disparities.

### 2.2. Lactoferrin Modulation of the Tuberculoid Granuloma

An examination of lactoferrin to modulate granulomatous responses was performed in mice injected intravenously with TDM. A subset of mice were intravenously given 1 mg bovine lactoferrin 24 h after TDM challenge. Lung tissue was analyzed for histological response and for the production of proinflammatory mediators. Treatment with lactoferrin showed statistically significant fewer and smaller granulomas compared to TDM alone [101]. Of importance, the protective proinflammatory mediators were not statistically diminished by lactoferrin treatment. Nontreated mice demonstrated a granuloma formation that correlated with an increased production of tumor necrosis factor-*α* (TNF-*α*), interleukin- (IL-) 1*β*, and IL-6. This is important, as it suggests that innate functions critical during infectious disease are maintained, even though immunopathology is reduced by treatment.

**Figure 3:**
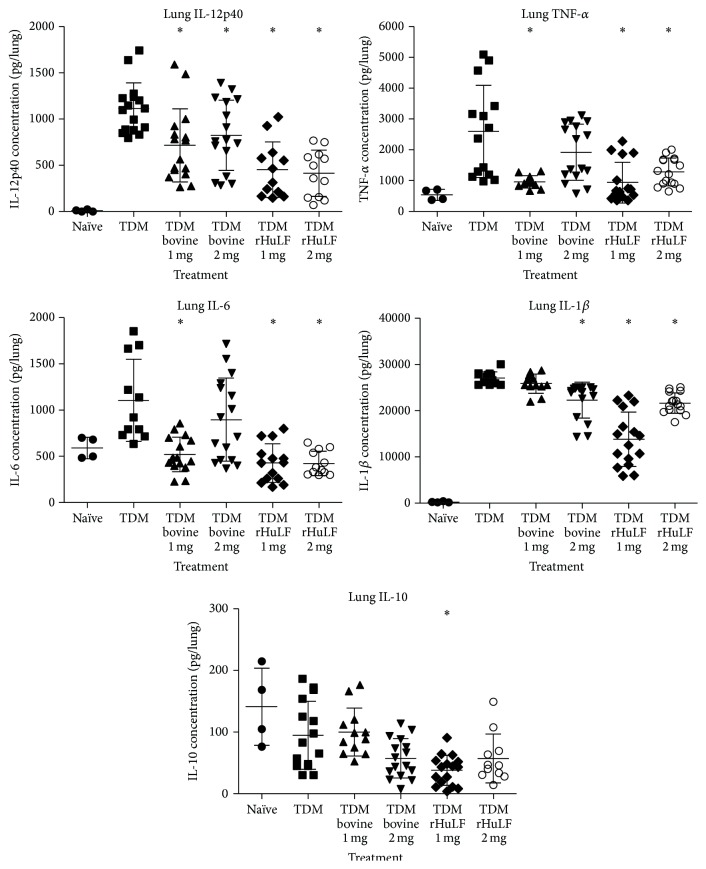
Proinflammatory cytokines in lungs are reduced in lactoferrin-treated mice after challenge with TDM. BALB/c mice were challenged with 25 *μ*g TDM in a water-in-oil emulsion. Bovine or recombinant human lactoferrin (1 or 2 mg) was orally administered at 4 and 6 days after TDM challenge. Methods of TDM induction are described elsewhere [38, 101]. Lungs were isolated on day 7 after TDM challenge; cytokines were assessed by ELISA; values are shown per lung for individual mice. ^*∗*^ *p* < 0.05 compared to the TDM emulsion alone treated mice.

In a similar manner, the utility of oral delivered lactoferrin was also examined. As previously mentioned, mice given TDM alone also showed marked and statistically significant increased production of proinflammatory cytokines TNF-*α*, IL-6, IL-1*β*, IL-12p40, and KC (keratinocyte chemoattractant). Oral treatment with recombinant human LF (produced in CHO cells) at 1 or 2 mg led to statistically significant reduction (*p* < 0.05) in cytokine levels at 7 days after TDM administration in BALB/c and C57BL/6 mice (Figure 3, only BALB/c data shown). This is consistent with reports that these proinflammatory cytokines are protective in similar infectious challenge models [102, 103], while molecules such as IL-10 which are limited after lactoferrin administration are detrimental during infection [104]. In a similar manner, bovine lactoferrin given orally at 1 or 2 mg doses also showed statistical reduction in most cytokines, although there was greater consistency of response in BALB/c mice than that for the C57BL/6 strain. In particular, the 1 mg dosage of recombinant LF showed the most consistency in its ability to control (reduce) production of proinflammatory cytokines.

**Figure 4:**
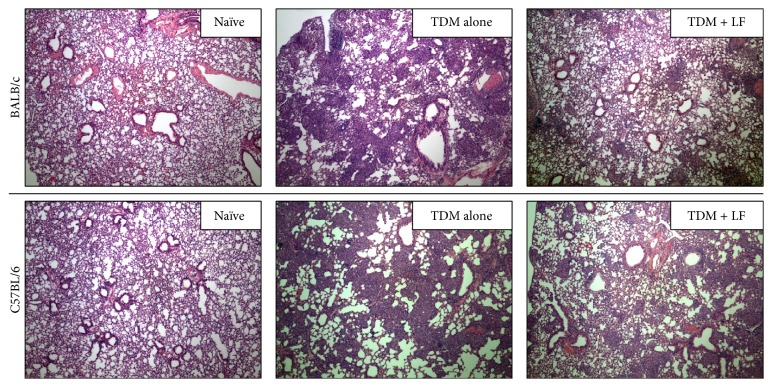
Granuloma response to TDM challenge in oral recombinant human lactoferrin-treated mice. Two strains of mice (BALB/c and C57BL/6) were challenged with 25 *μ*g TDM in a water-in-oil emulsion, as described [38, 101]. Human lactoferrin (recombinant, 1 mg) was given orally at 4 and 6 days after TDM challenge. Comparisons are made to naïve mice, and to mice only administered TDM. H&E staining, 100x. Representative sections from mice at 7 days after TDM challenge; *N* ≥ 8 per experimental group.

Of major interest, the oral delivered human recombinant lactoferrin demonstrated biologically relevant reduction in pathology (Figure 4), similar to that seen in the published results using intravenous administered bovine lactoferrin. In fact, both the bovine and the recombinant human lactoferrins were able to reduce inflammation due to TDM challenge, with doses of 1 or 2 mg given on days 4 and 6 after challenge resulting in clear reduction in granuloma size and frequency. The most effective dose for the recombinant human lactoferrin was seen when used at 1 mg, with some efficacy also seen given as a single dose only on day 4 (data not shown).

Another point of special interest is that the classical cytokines critical for control of MTB, namely, TNF-*α*, IL-6, and IFN-*γ*, were not statistically altered by lactoferrin treatment [105]. This gave confidence that treatment would retain control of organisms after infectious challenge. Indeed, this is what was seen in a similar experiment performed using bovine lactoferrin administered in drinking water (5 mg/mL), given to mice aerosol infected with virulent mycobacteria (Erdman strain) [105]. While bacterial load in tissue was slightly reduced, the major change was amelioration of granulomatous severity. It is noteworthy that bovine lactoferrin reduced bacterial burden, accompanied by an increase in classical proinflammatory responses while decreasing overall lung immunopathology [105]. Specifically, lactoferrin-treated mice increased numbers of CD4+ IFN-*γ*+ and IL-17 producing cells in the lung. It was shown that lactoferrin by itself was not bactericidal, but rather enhanced IFN-*γ* mediated MTB killing by macrophages in a nitric oxide dependent manner. These studies indicate that lactoferrin may be a novel therapeutic for the treatment of tuberculosis and may be useful in infectious diseases to reduced immune-mediated tissue damage.

Finally, the molecular mechanism underlying resolution of the granulomatous lesion is not yet defined. However, a role for maintaining strong Th1 responses could possibly be attributed to relationships due to COX-2 inhibition by lactoferrin in induced macrophages (WBCs) [106], and knowledge that prostaglandin E2 (PGE2) is able to shift T helper responses during BCG activation of innate macrophages [107]. It had been previously demonstrated that the PGE2 response could be reversed by a COX-2 inhibitor [108]. Therefore, lactoferrin functioning as a COX-2 inhibitor could be an indirect method to maintain proinflammatory activity of macrophages when infected.

## 3. Conclusions

The studies here indicate that lactoferrin may be a useful adjunct therapeutic for amelioration of pathological response in an* in vivo* model of TB granulomas. Of high importance, the change in pathology is accomplished with no loss of innate immune function which would be critical for defense against pathogenic organisms. A question arises concerning the utility of using heterologous lactoferrins in the mouse model. Preliminary investigations show that a newly developed, recombinant mouse lactoferrin produced in CHO stable cell lines functions in a manner very similar to the bovine and human recombinant, to limit immunopathology after oral delivery. Mechanistically, it was shown that the mouse and human forms are nearly identical in modulation of dendritic cell function in response to Bacillus Calmette-Guerin (BCG) [25, 109]. The dendritic cell population responds with increased inflammatory cytokines and a shift towards MHC Class II expression when BCG is combined with the lactoferrins. However, more recent investigations reveal that the macrophage population has a unique trend, with potentially decreased antigen presentation and subsequent T-cell stimulatory activity [110]. Overall, these results give great confidence to move forward to test novel recombinant lactoferrins for adjunct clinical therapeutic effects to alter immune dependent granulomatous pathology occurring during tuberculosis infection in humans. The studies here would drive the next goals to explore novel recombinant lactoferrins to mediate reductions in lung histopathology and bacterial burden in BSL3* in vivo* models of MTB infection. Furthermore, we hypothesize that reduction of TB-induced inflammatory pathology would also allow enhanced penetration of antimycobacterial drugs to sites harboring infectious agents, in essence permitting an efficient mechanism to treat TB infection. Overall, these studies permit confidence to target clinical implications and test novel strategies to modulate MTB-induced granulomatous responses to allow more efficacious delivery of therapeutics to mycobacterial organisms residing within lung tissue during active mycobacterial disease states.
